# Cardiovascular Risk Estimation Based on Country-of-Birth- and Country-of-Residence-Specific Scores among Migrants in the Netherlands: The HELIUS Study

**DOI:** 10.3390/ijerph20065148

**Published:** 2023-03-15

**Authors:** James Osei-Yeboah, Eric P. Moll van Charante, Andre-Pascal Kengne, Ellis Owusu-Dabo, Bert-Jan H. van den Born, Henrike Galenkamp-van der Ploeg, Felix P. Chilunga, Daniel Boateng, Ehsan Motazedi, Charles Agyemang

**Affiliations:** 1 Department of Public and Occupational Health, Amsterdam UMC, Amsterdam Public Health Research Institute, University of Amsterdam, Meibergdreef 9, 1012 WX Amsterdam, The Netherlands; 2 Department of Global and International Health, School of Public Health, Kwame Nkrumah University of Science and Technology, PMB KNUST, Kumasi GPS AK-448-4944, Ghana; 3 Department of General Practice, Amsterdam UMC, Amsterdam Public Health Research Institute, University of Amsterdam, Meibergdreef 9, 1012 WX Amsterdam, The Netherlands; 4 Non-Communicable Disease Research Unit, South African Medical Research Council, Cape Town 7505, South Africa; 5 Department of Vascular Medicine, Amsterdam Cardiovascular Sciences, Amsterdam UMC, University of Amsterdam, 1012 WX Amsterdam, The Netherlands; 6 Department of Epidemiology & Biostatistics, School of Public Health, Kwame Nkrumah University of Science and Technology, PMB KNUST, Kumasi GPS AK-448-4944, Ghana; 7 Julius Global Health, Julius Center for Health Sciences and Primary Care, University Medical Centre, 3584 CX Utrecht, The Netherlands; 8 Amsterdam UMC Location Vrije Universiteit Amsterdam, Public and Occupational Health, De Boelelaan 1117, 1081 HZ Amsterdam, The Netherlands

**Keywords:** cardiovascular risk scores, migrant populations, country-of-birth-specific scores, country-of-residence-specific scores

## Abstract

Background: Regional and country-specific cardiovascular risk algorithms have been developed to improve CVD risk prediction. But it is unclear whether migrants’ country-of-residence or country-of-birth algorithms agree in stratifying the CVD risk of these populations. We evaluated the risk stratification by the different algorithms, by comparing migrant country-of-residence-specific scores to migrant country-of-birth-specific scores for ethnic minority populations in the Netherlands. Method: data from the HELIUS study was used in estimating the CVD risk scores for participants using five laboratory-based (Framingham, Globorisk, Pool Cohort Equation II, SCORE II, and WHO II) and three nonlaboratory-based (Framingham, Globorisk, and WHO II) risk scores with the risk chart for the Netherlands. For the Globorisk, WHO II, and SCORE II risk scores, we also computed the risk scores using risk charts specified for the migrant home country. Risk categorization was first done according to the specification of the risk algorithm and then simplified to low (green), moderate (yellow and orange), and high risk (red). Results: we observed differences in risk categorization for different risk algorithms ranging from 0% (Globorisk) to 13% (Framingham) for the high-risk category, as well as differences in the country-of-residence- and country-of-birth-specific scores. Agreement between different scores ranged from none to moderate. We observed a moderate agreement between the Netherlands-specific SCORE II and the country-of-birth SCORE II for the Turkish and a nonagreement for the Dutch Moroccan population. Conclusion: disparities exist in the use of the country-of-residence-specific, as compared to the country-of-birth, risk algorithms among ethnic minorities living in the Netherlands. Hence, there is a need for further validation of country-of-residence- and country-of-birth-adjusted scores to ascertain appropriateness and reliability.

## 1. Introduction

Cardiovascular diseases (CVD) account for a large proportion of mortality, morbidity, and disability worldwide [1,2]. Primary prevention of CVD has lately moved towards the concept of ‘long-term’ risk detection of apparently healthy individuals for timely intervention to help prevent or delay the progression of the disease [3,4]. The use of mathematical equations (models) serves as tools to convert data on multiple risk factors into a summary estimate of a person’s likelihood of experiencing a cardiovascular event over a given period. Such risk scores have been very useful in early CVD risk detection and the start of preventive interventions [5,6]. The literature is inundated with various CVD risk prediction models, mostly derived from European and North American populations with varying thresholds and weights for component risk factors and definitions of CVD outcomes [7,8,9]. Despite the difference in make and form, all CVD risk scores categorized an individual’s risk of developing CVD from low to high.

The incidence of CVD and its’ impact on different ethnic groups varies as each group has a unique risk profile [10]. The predictive performance of CVD risk algorithms is therefore said to be best among the population in which it was derived and/or validated. This has given rise to regional- and country-specific algorithms developed mainly through the calibration and adjustment of existing models with population- and country-specific data. For migrants living in western countries, their risk prediction is often based on algorithms that are merely developed and validated for the host population, whereas those developed and validated for their country-of-birth compatriots might yield more appropriate or adequate risk estimations. There is no literature on the agreement or otherwise between the country-of-residence- and the country-of-birth-specific risk scores for minority populations living in western countries.

Using a population-based, multiethnic cohort, including populations of Dutch, Ghanaian, South-Asian Surinamese, African Surinamese, Moroccan, and Turkish ethnic origin peoples living in the Netherlands, this study aims to assess agreement between different CVD algorithms and compare the agreement between the use of algorithms from migrants’ country-of-birth-specific scores to country-of-residence- specific scores and the potential impact on these populations.

## 2. Methods

### 2.1. Study Population

The Healthy Life in an Urban Setting (HELIUS) study, consists of a multiethnic cohort of different ethnic groups living in Amsterdam. The aim of the HELIUS study is to gain insight into the causes of the unequal burden of disease across ethnic groups and ultimately enable the improvement of health care and prevention strategies. Full detail of the rationale, conceptual framework, design, and methods as well as the explanatory mechanisms being studied in the HELIUS study has been published in Stronks, et al. [11]. In brief, the HELIUS study includes adult populations of ethnic Dutch, Ghanaian, South-Asian Surinamese (Hindustani), African Surinamese (Creole), Moroccan, and Turkish origin. For the purposes of this paper, 13,794 participants aged 40 to 70 years, with complete data on ethnicity, biologic samples, physical examination, and questionnaire administration were drawn from the HELIUS data set. For this study, the population of second-generation migrants included was very small (271) and therefore did not confound the results for the country-of-birth scores.

### 2.2. Measurements of Cardiovascular Risk Score Estimation

CVD risk scores for participants were estimated using eight CVD risk algorithms. The CVD algorithms included in this study were five laboratory-based and three nonlaboratory-based equations. The laboratory-based equations tested were the Framingham [12], the Globorisk [13], the Reversed Pool Cohort Equation 2018 (PCE II) [14], SCORE II [15], and WHO II [16]. The nonlaboratory-based equations tested in this study were, the Framingham [12], the Globorisk [13], and the WHO II [16].

The laboratory-based Framingham CVD risk algorithm uses age, sex, HDL cholesterol, total cholesterol, diabetes status, smoking, and systolic blood pressure with treated or untreated hypertension as risk variables to compute a percentage risk score ranging from <1% to >30%. In the nonlaboratory-based/office Framingham CVD risk algorithm, HDL cholesterol and total cholesterol are replaced with BMI. Risk < 10% is classified as a low 10-year risk of developing CVD, 10 to 20% is intermediate/moderate, and >20% is a high risk of developing a CVD event [12].

The risk variables in the computation of the CVD score using the Globorisk algorithm are age, sex, total cholesterol level, diabetes status, smoking, and systolic blood pressure. Total cholesterol is replaced with BMI for the nonlaboratory/office-based Globorisk algorithm. The Globorisk uses a percentage base score that categorizes individuals into seven risk groups colour code as <5% deep green, 5 to 9% light green, 10 to 19% yellow, 20 to 29% orange, 30 to 39% light red, 40 to 49% deep red, and ≥50% dark red [13].

The Reversed Pool Cohort Equation 2018 uses age, sex, HDL cholesterol, total cholesterol, diabetes status, smoking, systolic blood pressure with or without the treatment of hypertension, and race as risk variables to compute a percentage risk score. One of the major differences between the Framingham and PCE is the introduction of race as a factor in the PCE. The risk of an individual developing a cardiovascular event in 10 years is categorized as <5% low, 5 to 7.4% borderline, 7.5 to 19.9% Intermediate, and ≥20% High risk [14].

The risk variables used for the computation of CVD risk in the SCORE II are age, sex, non-HDL cholesterol, smoking status, and systolic blood pressure. The categorization for the risk of developing a CV event in 10 years for individuals <50 years is <2.5% low/green, 2.5 to 7.5% moderate/mauve, and ≥7.5% high/red. For persons between the ages of 50 to 69 years, the risk categorization is <5% low/green, 5 to <10% moderate/mauve, and ≥10% high/red [15].

The WHO II CVD risk chart uses age, sex, total cholesterol, diabetes status, smoking, and systolic blood pressure as risk variables to compute a percentage risk score. The risk score is then adjusted based on the regional risk of developing CVD based on the Global Burden of Disease. Total cholesterol is replaced with BMI for the nonlaboratory/office-based WHO II algorithm. The risk level categorization is <5% green, 5% to <10% yellow, 10% to <20% orange, 20% to <30% red, and ≥30% dark red [16].

### 2.3. Statistical Analysis

Using IBM SPSS version 26, we calculated the absolute risk of developing CVD in 10 years for all participants. This was done by writing syntax from the CVD risk charts for each risk algorithm. For the Globorisk, WHO II, and SCORE II algorithms, we used the risk chart specified for the population living in the Netherlands for the entire study population (country-of-residence score). Additionally, we used the risk charts specified for the populations living in Ghana, Morocco, Suriname, and Turkey to calculate the country-of-birth score for each migrant population, respectively. Individuals’ risk of developing CV events was ranked according to the categorization assigned to the scores by each of the used risk algorithms. Harmonization of the rankings was achieved by classifying all green colour codes, i.e., low and borderline risks, as low risk, and all yellow and orange colour codes, i.e., intermediate/moderate risks, as moderate risk, and all the different shades of red colour codes, representing high to very high-risk ranks, were classified as high risk. We used Pearson’s Chi-square test to compare the distribution of CVD risk categories between ethnic groups. Agreement between different risk assessment algorithms, as well as between the use of the country-of-residence- and the country-of-birth-specific risk charts among the same ethnic group was assessed using Cohen’s kappa coefficient.

## 3. Results

The study population included 13794 participants of the HELIUS study, with an average age of 52.5 years (SD = 7.6) and 56.9% being female. This multiethnic population was made up of 2960 (21.5%) ethnic Dutch, 1684 (12.2%) African Surinamese, 3090 (22.4%) South-Asian Surinamese, 2073 (15.0%) Moroccan, and 1977 (14.3%) Turkish individuals living in the Netherlands. The rates of hypertension and diabetes were 24.8%, and 16.0%, respectively, and 22.6% of participants reported smoking at the time of data collection (Table 1).

Among the general study population, the percentage of people categorized as having a high risk of developing CVD ranged from 0% (Globorisk) to 13.0% (Framingham). Irrespective of the risk assessment algorithm used, the male participants were more frequently assigned to the high-risk category of CVD than the female participants (Table 2 and Table S1).

We observed ethnic disparities in the risk of developing CVD. The two Surinamese groups contained the largest percentage of high-risk individuals, followed by the ethnic Dutch, while the smallest percentage of high-risk individuals was observed among the Moroccans (Table 3).

For the three nonlaboratory-based CVD risk scores, the percentage of estimated high-risk individuals ranged from 0% (Globorisk) to 21.6% (Framingham). A larger percentage of high-risk individuals was observed among the male population compared to the female (Table 4 and Table S2).

Using the nonlaboratory-based CVD risk scores, significant ethnic disparities in risk estimates were observed for the Framingham and the WHO scores. The Surinamese population presented the highest risk of developing CVD, with the African Surinamese having the highest risk (Table 5).

**Figure 1:**
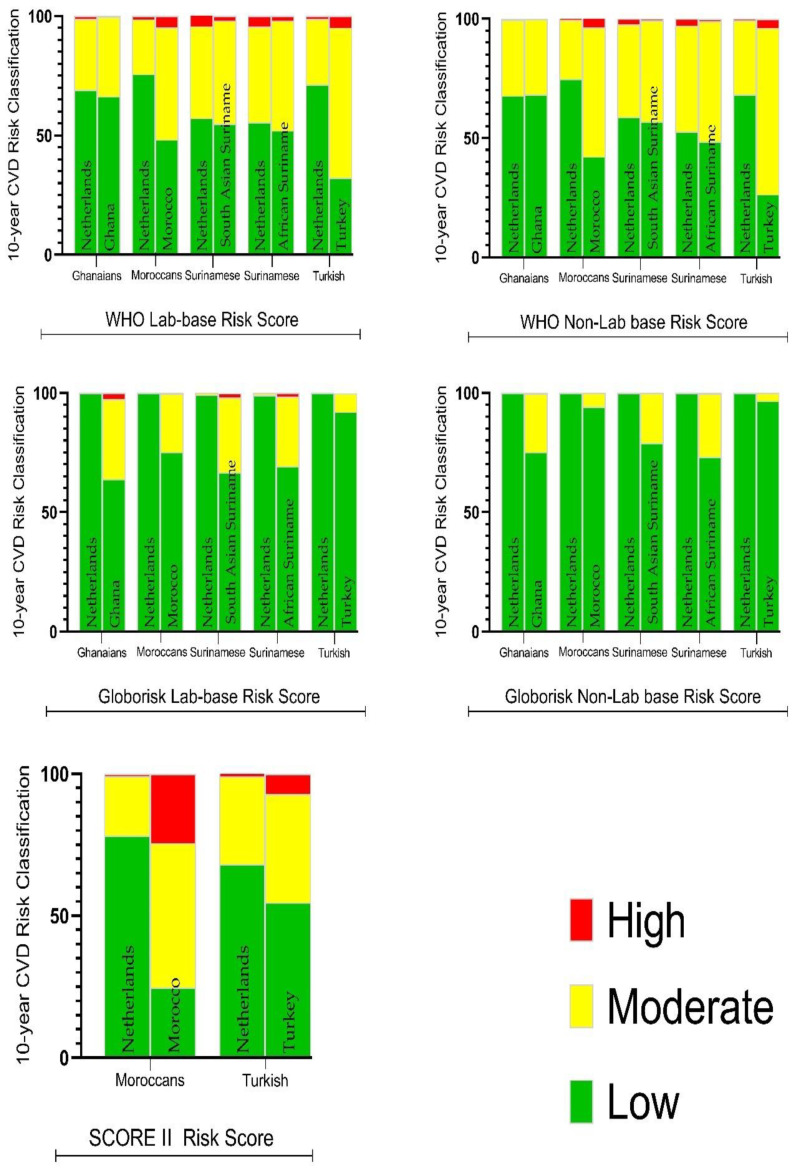
Percentage CVD Risk Classification among a multiethnic population living in the Netherlands, using the Netherlands/Dutch versus country-of-birth cardiovascular-risk scores. High = High risk CVD, Moderate = Moderate risk CVD, Low risk of CVD.

In general, the use of the migrant country-of-birth-specific algorithms was seen to categorize migrants at a higher risk of developing CVD compared to the country-of-residence-specific risk algorithms (The Netherlands). The difference in categorization between the country-of-residence scores and the country-of-birth scores was significant among the Moroccan and the Turkish population for the SCORE II and the WHO II algorithms. For the Ghanaian and the Surinamese populations, the differences in risk classification between the country-of-birth-specific and country-of-residence-specific algorithms were more profound when the Globorisk was used (Figure 1).

In general, the agreement between scores was weak. The SCORE II and the WHO II showed moderate agreement. The agreement between the laboratory-based models and their corresponding nonlaboratory-based models was moderate (Table 6 and Table S3).

The WHO lab-based model exhibited a moderate agreement between the Ghanaian as well as Surinamese-specific scores when compared with the Netherlands-specific score, though the agreement was weak among the Moroccans and minimal among Turkish. The WHO nonlaboratory model exhibited a strong agreement between the Ghana-specific score and the Netherlands-specific score, a moderate agreement among the Surinamese, and a minimal agreement among the North African migrant. The country-of-birth-specific SCORE II showed a moderate agreement with the Netherlands SCORE II among the Turkish, though no agreement among the Moroccans (Table 7, Tables S4 and S5).

## 4. Discussion

In this work, we focused on differences in agreement between the commonly used CVD prediction models in a diverse multiethnic population. We observed disagreement between different risk algorithms in the risk categorization. Also, for the same risk algorithm, there were disparities in estimated CVD risk among different ethnic groups and there were disagreements between the use of country-of-birth- and country-of-residence-specific scores.

Across the different ethnic groups, it could be said that a person’s estimated risk of CVD would depend on the CVD algorithm used. Though most models in recent times have been calibrated and refitted [13,14,15,16], large differences in risk estimates between models persist as earlier observed in the work of Wagner, et al. [17]. This observation has clinical implications for statin therapy [18].

The ethnic disparities in CVD risk observed are a reinforcement of the earlier report by Perini, et al. [19], who reported disparity by ethnicity in CVD risk classification by different CVD risk algorithms in this population. This could be explained in part by the difference in unique risk profiles among different ethnic groups [10]. Though inhabiting the same geographical space with common risks exposure, different ethnic groups, especially the migrant populations, appear to have different risk profiles compared to the host population and this may influence the impact of CVD on these migrant groups. For example, in this cohort, while smoking is common among those of Dutch and Turkish descent, it is rare among Ghanaians. Also, there is a significant difference in the prevalence of hypertension and diabetes among the various ethnic subgroups. Also to consider, are the issues of migration-related lifestyle changes, psychosocial stress, and low socioeconomic status, as well as discrepancies in genetic susceptibility and gene-environment interactions [20]. The early onset of CVD has been reported among some migrant groups, which may suggest that ethnic disparities in CVD may occur at a younger age [19].

Significantly higher estimates of the 10-year CVD risk were observed for migrants when the country-of-birth-specific models were used for the SCORE II, WHO II, and Globorisk scores. Country-specific scores are adjusted with country and regional population-specific characteristics such as the prevalence of CVD, CVD mortality, and the health status of a country, which are more of a representation of the majority population living in the country or region [13,14,15,16]. Thus, while mortality has decreased significantly due to the advent of acute interventions and preventive measures in developed countries, this cannot be said in developing countries. For minority populations living in such western societies, the question is how they benefit from such interventions. In the Netherlands, Agyemang, et al. [21], reported higher mortality after a first episode of CVD among ethnic minority patients than Dutch patients and suggested that treatment and secondary prevention strategies may be less effective among this group. The reverse was, however, observed in Denmark, with all-cause and cause-specific survival after CVD similar or significantly better for migrants compared to Danish-born [22]. The question that arises for migrant/minority populations living in western countries is, which of the two scores best estimate their CVD risk. Using the country-of-residence scores may lead to an underestimation of CVD risk, whiles using the country-of-origin score may yield an overestimation of this risk. This leads to the dilemma of the underestimation of migrant CVD risk with the use of the country-of-residence score or overestimation with a country-of-birth score.

It needs to be taken into account that the effect of genetic predisposition and a migrant’s subculture may lead to differential exposure to CVD risk for migrants compared to the host population [20]. It may be argued that using a country-of-birth-specific score may be more representative. However, there is the issue of cultural assimilation, which postulates that the longer people live away from their country of origin, the more likely they are to be oriented toward the host culture than that of their country of origin. In the current cohort, Perini, et al. [23] reported that neither the length of residence nor acculturation to the host culture influenced the risk of CVD. It may also be said that the combination of the above mentioned may lead to a subpopulation whose risk may neither be representative of their host country nor their country of birth. There is, therefore, the need for the validation of the existing models in such populations to achieve context-specific outcomes.

Our findings bring to the discussion policy choices for the treatment and prevention of CVD among ethnic migrant groups living in Western European societies. Since most treatment guidelines recommend the use of risk scores in the decision to initiate therapeutic and other interventions, it would be helpful for the validity of the risk score being used among migrant populations to be ascertained in order for such groups to benefit from such interventions.

### Strengths and Limitations

The strengths of our study lie in the large cohort which includes multiethnic migrant groups whose risk scores have not been assessed with their country-of-birth-specific risk scores and compared with their country-of-residence-specific risk scores. Thus, this may be a first in the literature. In the absence of datasets from the countries of birth, it is difficult to judge the overall comparability of the algorithms mentioned before, which is a major limitation in this analysis that needs to be acknowledged.

## 5. Conclusions

Disparities exist in the use of the country-of-residence-specific as compared to the country-of-birth risk algorithms among ethnic minorities living in the Netherlands. Hence, there is a need for further validation of country-of-residence- and country-of-birth-adjusted scores to ascertain appropriateness and reliability.

## Figures and Tables

**Table 1:** Demographic characteristics of the study population.

Parameter	Total	Percentage
All Participants	13,794	100
Age (years)	52.5	7.6
**Gender**		
Male	5950	43.1
Female	7844	56.9
**Ethnic Sub-Groups**		
Dutch	2960	21.5
Ghanaian	1684	12.2
African Surinamese	3090	22.4
South-Asian Surinamese	2073	15.0
Moroccan	2010	14.6
Turkish	1977	14.3
**Education Background**		
Unknown	144	1.0
Never or Elementary	3284	23.8
Lower Level	4203	30.5
Intermediate	3161	22.9
Higher/ University	3002	21.8
**CVD Risk Factors**		
Currently Smoking	3118	22.6
Hypertension	3417	24.8
Diabetic	2203	16.0
	Mean	SD
Systolic Blood Pressure (mmHg)	131.6	18.1
Total Cholesterol (mmol/L)	5.1	1.0
High-Density Lipoprotein (mmol/L)	1.5	0.4
Non-HDL Cholesterol (mmol/L)	3.7	1.0

Data is presented as a figure with a corresponding percentage or mean with standard deviation (SD).

**Table 2:** CVD Risk classification among multi-ethnic population living in the Netherlands using laboratory-based Cardiovascular risk scores.

Parameter	Framingham	Globorisk	PCE II	SCORE II	WHO II
**All participants**					
**Low**	12,014 (66.8)	19,779 (99.5)	16,239 (82.7)	13,366 (67.1)	12,976 (64.7)
**Moderate**	3668 (20.4)	98 (0.5)	2736 (13.9)	6051 (30.4)	6499 (32.4)
**High**	2337 (13.0)	0 (0.0)	672 (3.4)	515 (2.6)	577 (2.9)
**Male Participants**					
**Low**	3857 (50.0)	8442 (99.4)	5842 (69.6)	3560 (41.8)	3873 (45.3)
**Moderate**	1995 (25.8)	50 (0.6)	2018 (24.1)	4477 (52.6)	4243 (49.6)
**High**	1867 (24.2)	0 (0.0)	532 (6.3)	477 (5.6)	443 (5.2)
**Female Participants**					
**Low**	8157 (79.2)	11,337 (99.6)	10,397 (92.4)	9806 (85.9)	9103 (79.2)
**Moderate**	1673 (16.2)	48 (0.4)	718 (6.4)	1574 (13.8)	2256 (19.6)
**High**	470 (4.6)	0 (0.0)	140 (1.2)	38 (0.30)	134 (1.2)
***p* value**	<0.0001	0.101	<0.0001	<0.0001	<0.0001

Data are presented as the frequency with corresponding proportions in parenthesis. *p* is significant at 0.05 comparing the male and female 10-year risk of cardiovascular disease.

**Table 3:** CVD Risk classification among ethnic groups living in the Netherlands using laboratory-based cardiovascular risk scores.

Parameter	Framingham	Globorisk	PCE II	SCORE II	WHO II
**Dutch**					
Low	2562 (67.9)	4212 (99.7)	3598 (84.9)	2665 (62.9)	2643 (62.1)
Moderate	738 (19.6)	15 (0.4)	503 (11.9)	1406 (33.2)	1461 (34.3)
High	47 2(12.5)	0 (0.0)	135 (3.2)	165 (3.9)	151 (3.6)
**Ghanaian**					
Low	1427(70.4)	2104 (99.8)	1714 (80.4)	1653 (77.6)	1481 (69.0)
Moderate	427 (21.1)	4 (0.2)	367 (17.2)	463 (21.7)	638 (29.7)
High	172 (8.5)	0 (0.0)	50 (2.4)	15 (0.7)	27 (1.3)
**Moroccan**					
Low	2217 (76.1)	3332 (99.9)	3019 (90.4)	2613 (78.3)	2556 (76.2)
Moderate	461 (15.8)	3 (0.1)	276 (8.3)	699 (20.9)	752 (22.4)
High	235 (8.1)	0 (0.0)	46 (1.4)	27 (0.8)	46 (1.4)
**South Asian Surinamese**					
Low	1563 (57.9)	2939 (99.1)	2326 (78.2)	1800 (60.5)	1719 (57.6)
Moderate	616 (22.8)	28 (0.9)	477 (16.0)	1066 (35.8)	1137 (38.1)
High	523 (19.4)	0 (0.0)	172 (5.8)	108 (3.6)	128 (4.3)
**African Surinamese**					
Low	2114 (58.8)	3823 (99.0)	2865 (74.0)	2342 (60.5)	2189 (56.0)
Moderate	867 (24.1)	40 (1.0)	799 (20.6)	1371 (35.4)	1547 (39.6)
High	617 (17.2)	0 (0.0)	210 (5.4)	159 (4.1)	174 (4.5)
**Turkish**					
Low	1975 (72.1)	3081 (99.8)	2717 (87.9)	2105 (68.1)	2224 (71.5)
Moderate	487 (17.8)	5 (0.2)	314 (10.2)	955 (30.9)	847 (27.2)
High	277 (10.1)	0 (0.0)	59 (1.9)	29 (0.9)	39 (1.3)
***p* value**	<0.0001	<0.0001	<0.0001	<0.0001	<0.0001

Data are presented as the frequency with corresponding proportions in parenthesis; *p* is significant at 0.05 comparing the ethnic 10-year risk of cardiovascular disease.

**Table 4:** CVD risk classification among the population living in the Netherlands using nonlaboratory-based Cardiovascular risk scores.

Parameter	Framingham	Globorisk	WHO II
**All participants**			
**Low**	10,049 (55.2)	20,016 (99.9)	12,622 (63.0)
**Moderate**	4253 (23.4)	19 (0.1)	7086 (35.3)
**High**	3908 (21.5)	0 (0.0)	344 (1.7)
**Male Participants**			
**Low**	2939 (37.7)	8540 (99.8)	3615 (42.2)
**Moderate**	2160 (27.7)	14 (0.2)	4651 (54.4)
**High**	2703 (34.6)	0 (0.00)	293 (3.4)
**Female Participants**			
**Low**	7110 (68.3)	11,476 (99.96)	9007 (78.4)
**Moderate**	2093 (20.1)	5 (0.04)	2435 (21.2)
**High**	1205 (11.6)	0 (0.0)	51 (0.4)
***p* value**	<0.0001	0.006	<0.0001

Data are presented as the frequency with corresponding proportions in parenthesis; *p* is significant at 0.05 comparing the male and female 10-year risk of cardiovascular disease.

**Table 5:** CVD risk classification among ethnic groups living in the Netherlands using nonlaboratory-based Cardiovascular risk scores.

Parameter	Framingham	Globorisk	WHO II
**Dutch**			
Low	2263 (59.7)	4250 (99.9)	2564 (60.2)
Moderate	799 (21.1)	2 (0.1)	1586 (37.3)
High	729 (19.2)	0 (0.0)	105(2.5)
**Ghanaian**			
Low	1079 (52.5)	2143 (100)	1455 (67.8)
Moderate	564 (27.5)	0 (0.0)	681 (31.7)
High	411 (20.0)	0 (0.0)	10 (0.5)
**Moroccan**			
Low	1938 (65.9)	3350 (99.9)	2508 (74.8)
Moderate	572 (19.5)	2 (0.1)	827 (24.6)
High	428 (14.6)	0 (0.0)	19 (0.6)
**South Asian Surinamese**			
Low	1329 (48.9)	2977 (99.8)	1754 (58.8)
Moderate	644 (23.7)	5 (0.2)	1159 (38.8)
High	747 (27.5)	0 (0.0)	71 (2.4)
**African Surinamese**			
Low	1607 (44.0)	33897 (99.8)	2059 (52.7)
Moderate	972 (26.6)	8 (0.2)	1737 (44.4)
High	1071 (29.4)	0 (0.0)	114 (2.9)
**Turkish**			
Low	1710 (61.5)	3107 (99.97)	2124 (68.3)
Moderate	620 (22.3)	1 (0.03)	966 (31.1)
High	451 (16.2)	0 (0.0)	20 (0.6)
*p* value	<0.0001	0.020	<0.0001

Data are presented as the frequency with corresponding proportions in parenthesis; *p* is significant at 0.05 comparing the male and female 10-year risk of cardiovascular disease.

**Table 6:** Agreement in cardiovascular risk classification between risk scores among a multiethnic population living in the Netherlands.

Parameter	Globorisk	PCE II	SCORE II	WHO II	Framingham NL	Globorisk NL	WHO II NL
Framingham	0.008 ***	0.432 ***	0.425 ***	0.576 ***	0.611 ***	0.001 **	0.509 ***
Globorisk		0.020 ***	0.012 ***	0.005 **	0.004 ***	0.155 ***	0.009 ***
PCE II			0.424 ***	0.515 ***	0.235 ***	0.004 ***	0.449 ***
SCORE II				0.598 ***	0.327 ***	0.001 *	0.655 ***
WHO II					0.463 ***	0.001	0.784 ***
Framingham NonLab						0.001	0.460 ***
Globorisk NonLab							0.001

Data is presented as Cohen’s kappa coefficients where 0–0.2 None, 0.21–0.39 minimal, 0.4–0.57 weak, 0.6–0.79 moderate, 0.8–0.90 strong, >0.9 almost perfect. *p* is significant at 0.05 *, 0.01 **, 0.001 ***. NonLab: Nonlaboratory base score.

**Table 7:** Agreement in cardiovascular risk classification among the Netherlands’ specific risk scores and the country-of-birth risk score within the individual ethnic groups.

Parameter	Globorisk	WHO II	SCORE II	Globorisk NL	WHO II NL
**Ghanaian**	0.002	0.780 ***	N/A	−0.023 ***	0.818 ***
**Moroccan**	0.000	0.404 ***	−0.004	0.019 ***	0.350 ***
**South Asian Surinamese**	0.014 **	0.751 ***	N/A	0.009 **	0.784 ***
**African Surinamese**	0.018 ***	0.763 ***	N/A	0.004	0.756 ***
**Turkish**	0.012 ***	0.279 ***	0.615 ***	0.018 ***	0.252 ***

Data is presented as kappa coefficients of agreement where 0–0.2 None, 0.21–0.39 minimal, 0.4–0.57 weak, 0.6–0.79 moderate, 0.8–0.90 strong, >0.9 almost perfect agreement. P is significant at 0.05 = *, 0.01 = **, and 0.001= ***. NL: Nonlaboratory base score.

## Data Availability

The used of data for this study is under license and not publicly available. Data request or any further question may be channeled to Henrike Galenkamp, the Data Coordinator of the HELIUS study (h.galenkamp@amsterdamumc.nl).

## Supplementary Materials

The following supporting information can be downloaded at: https://www.mdpi.com/article/10.3390/ijerph20065148/s1, Table S1: The 90th percentile of risk scores among a multi-ethnic population living in the Netherlands using the laboratory to based CVD risk Scores; Table S2: The 90th percentile of risk scores among a multi-ethnic population living in the Netherlands using Nonlaboratory based CVD risk Scores; Table S3: Agreement in cardiovascular risk classification between risk scores among a multi-ethnic population living in the Netherlands using the 90th percentile; Table S4: The 90th percentile of risk for the Netherlands specific risk algorithm and country of birth specific algorithm among a multi-ethnic population living in the Netherlands; Table S5: Agreement in cardiovascular risk classification among the Netherlands-specific risk scores and the country of birth risk score using the 90th percentile.
