# Supplementary material for: Cardiovascular Risk Estimation Based on Country-of-Birth- and Country-of-Residence-Specific Scores among Migrants in the Netherlands: The HELIUS Study
Source: Int J Environ Res Public Health. 2023 Mar 15;20(6):5148. doi: 10.3390/ijerph20065148 (PMC10048928; doi:10.3390/ijerph20065148)
Supplement: Supplementary file 1 [file ijerph-20-05148-s001.zip › ijerph-2224720-supplementary.pdf]

Supplementary File:

Table S1:The 90th percentile of risk scores among a multi-ethnic population living in the Netherlands using the laboratory to based CVD risk Scores

Parameter	Framingham	Globorisk	PCE II	SCORE II	WHO II
**Total**	1738(9.65)	1333(6.71)	1453(17.31)	1339(6.72)	1910(9.65)
**Gender**					
**Male**	1416(18.34)	864(10.17)	402(3.57)	1136(13.34)	1513(17.86)
**Female**	322(3.13)	469(4.12)	1855(9.44)	203(1.78)	397(3.50)
**p value**	<0.0001	<0.0001	<0.0001	<0.0001	<0.0001
**Ethnicity**					
**Dutch**	344(9.12)	247(5.84)	354(8.36)	433(10.22)	514(12.17)
**Ghanaian**	111(5.48)	92(4.36)	195(9.15)	64(3.00)	135(6.41)
**Moroccan**	164(5.63)	124(3.72)	158(4.73)	103(3.08)	161(4.85)
**South Asian Surinamese**	423(15.66)	327(11.02)	404(13.58)	251(8.44)	387(13.11)
**African Surinamese**	465(12.92)	378(9.79)	564(14.56)	375(9.68)	518(13.49)
**Turkish**	200(7.30)	143(4.63)	180(5.83)	87(2.82)	161(5.25)
**p value**	<0.0001	<0.0001	<0.0001	<0.0001	<0.0001

Data are presented as the frequency with corresponding proportions in parenthesis. p is significant at 0.05 comparing the male and female 10 to year risk of cardiovascular disease.

Table S2:The 90th percentile of risk scores among a multi-ethnic population living in the Netherlands using Nonlaboratory based CVD risk Scores

Parameter	Framingham	Globorisk	WHO II
**Total**	2311(12.69)	1458(7.28)	1809(9.02)
**Gender**			
**Male**	1797(23.03)	1084(12.67)	1387(16.21)
**Female**	514(4.94)	374(3.26)	422(3.67)
**p-value**	<0.0001	<0.0001	<0.0001
**Ethnicity**			
**Dutch**	449(11.84)	378(8.89)	496(11.66)
**Ghanaian**	220(10.71)	110(5.13)	110(5.13)
**Moroccan**	230(7.83)	107(3.19)	146(4.35)
**South Asian Surinamese**	472(17.35)	252(8.45)	312(10.46)
**African Surinamese**	656(17.97)	455(11.65)	553(14.14)
**Turkish**	245(8.81)	131(4.21)	158(5.08)
**p-value**	<0.0001	<0.0001	<0.0001

Data are presented as the frequency with corresponding proportions in parenthesis. p is significant at 0.05 comparing the male and female 10-year risk of cardiovascular disease.

Table S3: Agreement in cardiovascular risk classification between risk scores among a multi-ethnic population living in the Netherlands using the 90th percentile

Parameter	Globorisk	PCE II	SCORE II	WHO II	Framingham NL	Globorisk NL	WHO II NL
Framingham	0.635***	0.775***	0.599***	0.709***	0.582***	0.582**	0.632***
Globorisk		0.685***	0.518***	0.740***	0.627***	0.563***	0.561***
PCE II			0.571***	0.734***	0.788***	0.602***	0.641***
SCORE II				0.686***	0.572***	0.734*	0.778***
WHO II					0.722***	0.637***	0.714***
Framingham NL						0.599***	0.677***
Globorisk NL							0.825***

Data is presented as kappa coefficients of agreement where 0 to 0.2 None, 0.21 to 0.39 minimal, 0.4 to 0.57 weak, 0.6 to 0.79 moderate, 0.8 to 0.90 strong, > 0.9 almost perfect. P is significant at 0.05*, 0.01**,0.001***. NL: Non to laboratory base score.

Table S4:The 90th percentile of risk for the Netherlands specific risk algorithm and country of birth specific algorithm among a multi-ethnic population living in the Netherlands

Parameter	Globorisk	SCORE II	WHO II	WHO II NL	Globorisk NL
**Ghanaian**					
Netherlands	178(8.49)	64(3.00)	191(9.12)	188(8.76)	110(3.13)
Country of birth	190(9.01)		167(7.93)	145(6.76)	194(9.05)
**Moroccans**					
Netherlands	110(5.13)	271(8.07)	289(8.78)	280(8.35)	306(9.13)
Country of birth	194(9.05)	312(9.34)	316(9.49)	291(8.68)	319(9.52)
**South Asian Surinamese**					
Netherlands	193(6.50)	251(8.44)	256(8.71)	260(8.71)	252(8.45)
Country of birth	256(8.63)		268(9.07)	284(9.52)	236(7.91)
**African Surinamese**					
Netherlands	378(9.79)	375(9.68)	341(8.91)	366(9.36)	227(5.81)
Country of birth	338(8.75)		333(8.66)	353(9.03)	376(9.63)
**Turkish**					
Netherlands	241(7.81)	299(8.87)	271(8.87)	260(8.36)	131(4.21)
Country of birth	246(7.97)	241(7.80)	303(9.86)	277(8.91)	222(7.14)

Data are presented as the frequency with corresponding proportions in parenthesis. NL: Non to laboratory base score.

Table S5: Agreement in cardiovascular risk classification among the Netherlands-specific risk scores and the country of birth risk score using the 90th percentile.

Parameter	Globorisk	WHO II	SCORE II	Globorisk NL	WHO II NL
**Ghanaian**	0.748***	0.798***	N/A	0.669***	0.776***
**Moroccan**	0.669***	0.809***	0.833***	0.652***	0.780***
**South Asian Surinamese**	0.771***	0.885***	N/A	0.888***	0.822***
**African Surinamese**	0.772***	0.869***	N/A	0.0.726***	0.858***
**Turkish**	0.735***	0.804***	0.805***	0.680***	0.778***

Data is presented as kappa coefficients of agreement where 0 to 0.2 None, 0.21 to 0.39 minimal, 0.4 to 0.57 weak, 0.6 to 0.79 moderate, 0.8 to 0.90 strong, > 0.9 almost perfect agreement. P is significant at 0.05*, 0.01**,0.001***. NL: Non to laboratory base score.
